# Book Reviews

**Published:** 1907-03

**Authors:** 


### BOOK REVIEWS.

THORNTON'S POCKET MEDICAL FORMULARY. New (8th) edition, revised to accord with the new U. S. Pharmacopoeia. Containing about 2,000 prescriptions with indications for their use. In one leather bound volume. Price, $1.50 net. Lea Brothers & Co., Publishers, Philadelphia and New York, 1907.

It would be difficult to mention a more frequently useful work than Thornton's Formulary. The author is peculiarly qualified to render such a service, as he unites in himself a knowledge of the three necessary branches, being a graduate in pharmacy, a professor of materia-medica in a leading medical college, and an active practitioner of many years' standing. He has here presented the collective experience of his profession as to the best measures for combating disease. He has arranged diseases alphabetically, and under each has given the best formulæ for simple cases, as well as for the various stages and complications, with quantities both in the ordinary and metric systems. A feature peculiar to this work, and one of obvious value, is found in the indications and annotations for a choice between the various formulæ according to the conditions to be met.

---

ESSENTIALS OF OBSTETRICS. By Charles Jewett, M. D., Professor of Obstetrics ad Gynecology, in the Long Island College Hospital, Brooklyn, N. Y. Third edition, thoroughly revised. 12mo, 413 pages, with 80 engravings and 5 colored plates. Cloth, $2.25 net. Lea Brothers & Co., Philadelphia and New York, 1907.

This compact volume is intended as an introduction to the more elaborate treatises, and as a guide in following the didactic and practical teaching of college courses. Most attention has been given to practical topics. Works of this character have their distinct place and value, since mastery of the elements of any subject gives the rational frame-work for an easy and orderly acquisition of complete and systematic knowledge. Such a work is, therefore, useful not only to the student but also to the practitioner who would refresh his recollection or post himself to date. That Professor Jewett has interested both classes of readers, is shown by the demand for this new (the third) edition. It has been completely revised, largely rewritten, and rounded out with much entirely new matter.

---

No case of hemorrhoids should be dismissed after merely an external examination. The possibility that the piles may be evidences of an obstruction higher up in the intestine or in the portal circulation must always be inquired into.—American Journal of Surgery.

---

A gradually increasing anemia in an elderly person, without any other symptoms, is highly suggestive of a latent carcinoma, often in the intestine.—American Journal of Surgery.

---

Before attributing enlargement of the liver to a surgical conditon exclude chronic hepatic congestion of cardiac disease.—American Journal of Surgery.

---

It is well to remember that not all ulcers of the stomach are characterized by the classical symptoms of pain, vomiting and hemorrhage. Many patients presenting "dyspeptic" symtpoms of only mild grade are afflicted with this disease and such cases may easily be diagnosed as functional disorders until the persistence of the symptoms leads one to suspect the graver malady.—American Journal of Surgery.

